# Apolipoprotein E3 and E4 isoforms exhibit differing effects in countering endotoxins

**DOI:** 10.1016/j.jbc.2025.108236

**Published:** 2025-01-27

**Authors:** Manoj Puthia, Jan K. Marzinek, Katerina Vesela, Axel Larsson, Artur Schmidtchen, Peter J. Bond, Jitka Petrlova

**Affiliations:** 1 Division of Dermatology and Venereology, Department of Clinical Sciences, Lund University, Lund, Sweden; 2 Bioinformatics Institute (BII), Agency for Science, Technology and Research (A∗STAR), Singapore, Republic of Singapore; 3 BIOCEV, First Faculty of Medicine, Charles University, Prague, Czech Republic; 4 Department of Paediatrics and Inherited Metabolic Disorders, First Faculty of Medicine, Charles University and General University Hospital in Prague, Prague, Czech Republic; 5 Dermatology, Skåne University Hospital, Lund, Sweden; 6 Department of Biological Sciences National University of Singapore, Singapore, Singapore; 7 Department of Biomedical Science, Faculty of Health and Society, Malmö University, Malmö, Sweden

**Keywords:** apolipoprotein E isoforms, protein aggregation, host defense, endotoxin, antimicrobial peptides

## Abstract

Apolipoprotein E (APOE) is distributed across various human tissues and plays a crucial role in lipid metabolism. Recent investigations have uncovered an additional facet of APOE's functionality, revealing its role in host defense against bacterial infections. To assess the antibacterial attributes of APOE3 and APOE4, we conducted antibacterial assays using *Pseudomonas aeruginosa* and *Escherichia coli*. Exploring the interaction between APOE isoforms and lipopolysaccharides (LPSs) from *E. coli*, we conducted several experiments, including gel shift assays, CD, and fluorescence spectroscopy. Furthermore, the interaction between APOE isoforms and LPS was further substantiated through atomic resolution molecular dynamics simulations. The presence of LPS induced the aggregation of APOE isoforms, a phenomenon confirmed through specific amyloid staining, as well as fluorescence and electron microscopy. The scavenging effects of APOE3/4 isoforms were studied through both *in vitro* and *in vivo* experiments. In summary, our study established that APOE isoforms exhibit binding to LPS, with a more pronounced affinity and complex formation observed for APOE4 compared with APOE3. Furthermore, our data suggest that APOE isoforms neutralize LPS through aggregation, leading to a reduction of local inflammation in experimental animal models. In addition, both isoforms demonstrated inhibitory effects on the growth of *P. aeruginosa* and *E. coli*. These findings provide new insights into the multifunctionality of APOE in the human body, particularly its role in innate immunity during bacterial infections.

Apolipoprotein E (APOE) is a glycoprotein consisting of 299 amino acids, with a pivotal role in the regulation of cholesterol and lipid levels in both the bloodstream and the brain. In the human population, the APOE gene displays genetic diversity, resulting in the presence of three main alleles located on chromosome 19: APOE2, APOE3, and APOE4. These isoforms differ exclusively in their amino acid composition at positions 112 and 158 (1). The most widespread variant is APOE3, found in 78% of the human population, characterized by cysteine at position 112 and arginine at position 158. Conversely, APOE2 (present in 8% of the population) features cysteine at both positions, whereas APOE4 (occurring in 14% of the population) possesses arginine at both sites (2).

The exchangeable apolipoprotein is a constituent of high-density lipoprotein, very low-density lipoprotein, and low-density lipoprotein remnant particles, all of which contribute to atherosclerosis. APOE's multifaceted role impacts a range of processes, encompassing cholesterol removal, coagulation, macrophage function, oxidative mechanisms, central nervous system physiology, cellular signaling, and inflammatory responses (3).

Notably, APOE-derived antimicrobial peptides (AMPs) from the receptor- and heparin-binding region have demonstrated antibacterial, antiviral, and immunomodulatory effects (4, 5). In addition, previous research has revealed the antibacterial activity of full-length APOE against Gram-negative bacteria both *in vitro* and *in vivo* (6, 7).

Gram-negative bacteria are known for their capacity to cause severe infections in humans. The lipopolysaccharide (LPS) in their outer membrane can induce a strong inflammatory response when released into the bloodstream, potentially leading to septic shock and systemic inflammatory response syndrome. These infections are a significant health concern because of their severity, the rise of multidrug-resistant strains, and their frequent association with health care–acquired infections, particularly in immunocompromised patients (8, 9).

While our comprehension of the precise role of lipid-poor or free APOE remains incomplete, there is a hypothesis suggesting that APOE may exert a neutralizing effect on Gram-negative bacterial infections. The objective of this study is to provide evidence supporting the antibacterial properties of APOE3 and E4 isoforms against Gram-negative bacterial strains in an *in vitro* setting. In addition, the study aims to identify and validate isoform-specific interactions and the formation of APOE aggregates in response to LPS using biophysical methods, microscopy, and molecular dynamics (MD) simulations. LPS, a toxic component of the Gram-negative bacterial cell envelope, can lead to septic shock when released into the bloodstream. Therefore, our research further explores the scavenging effects of APOE isoforms *in vitro* and *in vivo* during LPS challenge.

## Results

### Antibacterial activity of recombinant APOE3 and E4 isoforms *in vitro*

**Figure 1:**
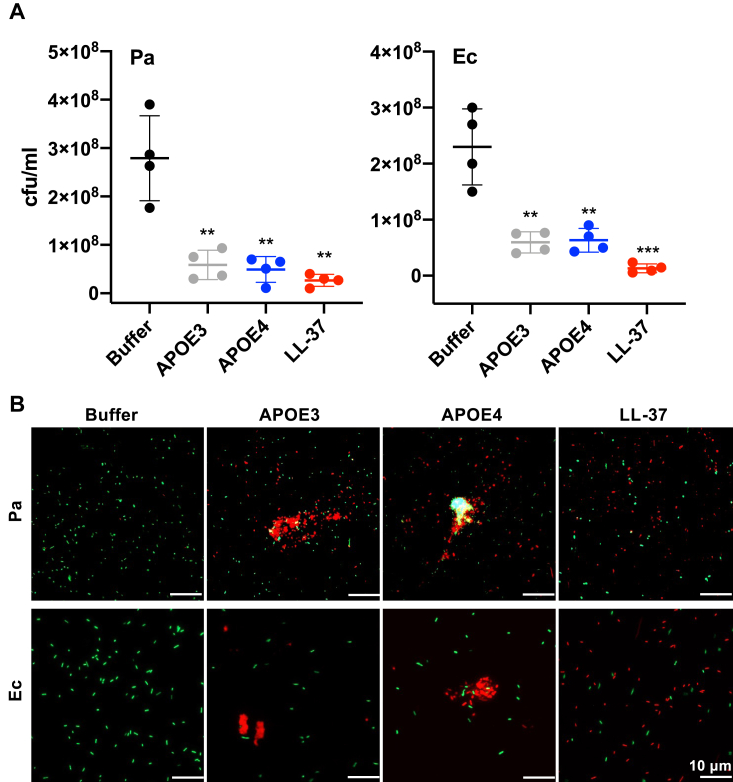
**Antimicrobial activity of APOE *in vitro*.***A*, *Escherichia coli* and *Pseudomonas aeruginosa* were incubated with 2 μM rAPOE isoforms for 2 h and then viable count assay was performed. LL-37 was used as a positive control, whereas bacteria treated with Tris buffer were used as a negative control. Data are presented as the mean ± SD of four independent experiments (n = 4). *B*, visualization of *E. coli* and *P. aeruginosa* viability. Live/dead viability assay of Gram-negative bacteria stimulated with 2 μM rAPOE, 10 mM Tris buffer at pH 7.4 (negative control), or 2 μM LL-37 (positive control). Representative images from four independent experiments are presented (n = 4). At least 10 individual fields were acquired per experiment. Live bacteria were stained with *green* SYTO 9 nucleic acid fluorescent dye, and dead bacteria were stained using red propidium iodine dye. Scale bar represents 10 μm. Statistical analysis was performed using a one-way ANOVA with Dunnett’s multiple comparison tests, ∗∗*p* ≤ 0.01, ∗∗∗*p* ≤ 0.001. APOE, apolipoprotein E; rAPOE, recombinant APOE3.

Recombinant APOE3 (rAPOE3) and rAPOE4 were obtained through purification from a bacterial expression system utilizing chemically competent *Escherichia coli*. Subsequently, their potential antimicrobial effects against two Gram-negative strains, *E. coli* and *Pseudomonas aeruginosa*, were assessed using the viable count assay (VCA), as shown in Figure 1*A*. Notably, a significant reduction in colony numbers for both *P. aeruginosa* and *E. coli* (approximately a twofold decrease in colony-forming unit/ml) was observed in samples treated with 2 μM rAPOE3 and rAPOE4. This concentration closely mirrors the protein's physiological levels in plasma, which typically range from 0.9 to 2 μM in healthy individuals (10). Furthermore, the bacterial killing efficiency of rAPOE isoforms against *P. aeruginosa* and *E. coli* was on par with that of the AMP LL-37, employed here as a positive control (11).

Furthermore, the VCA data findings were corroborated through the use of a live/dead imaging assay. The results unveiled the aggregation of killed bacteria when exposed to 2 μM of rAPOE3 and rAPOE4 isoforms, respectively (Fig. 1*B*). In this assay, dead bacteria with compromised membrane integrity appears in red, whereas live bacteria with intact membranes appears green.

### Structural changes of rAPOE isoforms upon endotoxin challenge

**Figure 2:**
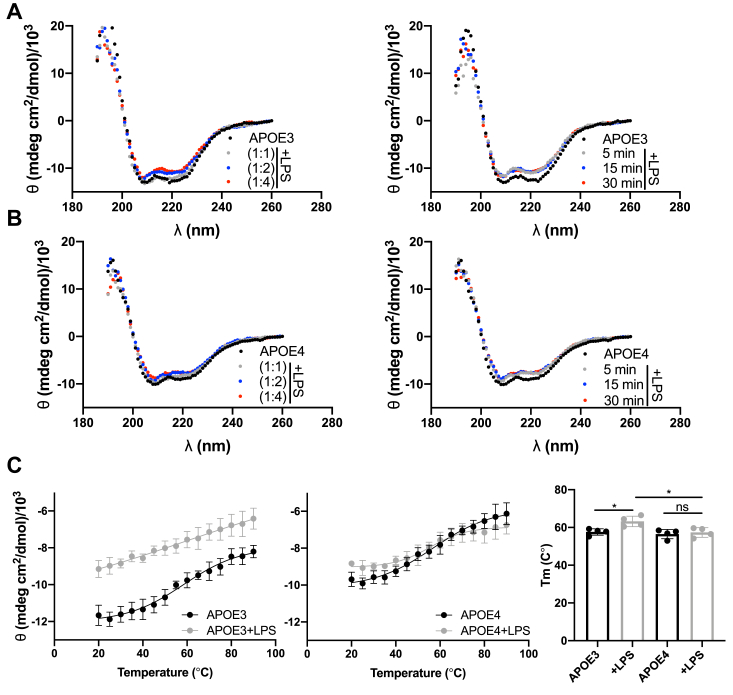
**Interaction with LPS induces a change in the secondary structure of APOE.** Far-UV CD spectra depict 5 μM rAPOE3 (*A*) and rAPOE4 (*B*) in isolation or following incubation with LPS at molar ratios of 1:1, 1:2, and 1:4. For a time-dependent experiment, the molar ratio 1:2 was utilized. Representative spectra from a minimum of three independent experiments (n ≥ 3) are presented. *C*, the melting temperature was determined from the temperature-dependent changes in the CD spectra at 222 nm. Statistical analysis employed a one-way ANOVA with Dunnett’s multiple comparison tests, where ∗ indicates *p* ≤ 0.05, ∗∗ denotes *p* ≤ 0.01, and ns signifies not significant. APOE, apolipoprotein E; LPS, lipopolysaccharide, rAPOE, recombinant APOE.

We conducted far-UV CD spectroscopy to record ellipticity spectra of rAPOE isoforms (at a concentration of 5 μM) both in isolation and in combination with varying concentrations and incubation times with endotoxin (LPS from *E. coli*). This allowed us to monitor changes in the secondary structure of proteins. The data obtained revealed a decrease in the α-helical content of both rAPOE isoforms upon their interaction with the ligands, as illustrated in Figure 2, *A* and *B*.

To further explore the complex stability between APOE isoforms and bacterial endotoxins, we conducted an additional experiment in which we examined the heat stability of the proteins by measuring ellipticity signals at 222 nm from temperature 25 to 90 °C. The melting temperature was similar for both rAPOE isoforms (rAPOE3 *T*_m_ = 57.6 ± 1.7 °C and rAPOE4 *T*_m_ = 56.5 ± 2.4 °C) but significantly higher in the presence of LPS and APOE3 (APOE3 *T*_m_ = 63.2 ± 2.8 °C and APOE4 57.4 ± 2.6 °C). The melting points were calculated using a Boltzmann fit (Figs. 2*C* and S1).

### Interaction of rAPOE isoforms with endotoxin

**Figure 3:**
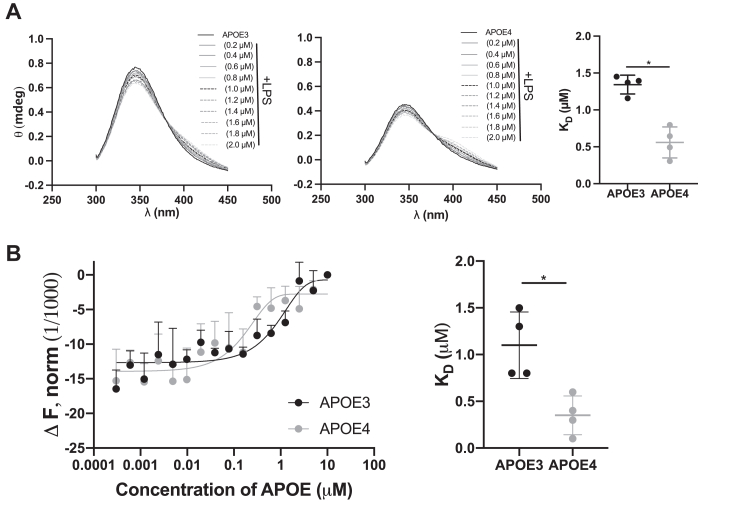
**Structural property of APOE is affected by interaction with bacterial products.***A*, increasing concentrations of LPS from *Escherichia coli* was added to 5 μM APOE, and then, intrinsic fluorescence spectra were recorded. Figures show representative spectra from four independent experiments (n = 4). *B*, dissociation constants (*K*_*D*_) of protein–ligand interaction were calculated from the binding curves. ∗*p* < 0.05. *C*, the MST binding curves were obtained reporting the mobility change with the increasing concentration of LPS-FITC (0.3 nM–10 μM) in the presence of APOE isoforms. Dissociation constants (*K*_*D*_) of protein–ligand interaction were calculated from the binding curves. Statistical analysis was performed using one-way ANOVA with Dunnett’s multiple comparison tests from four independent experiments (n = 4). ∗*p* < 0.05 and ns = not significant. APOE, apolipoprotein E; LPS, lipopolysaccharide; MST, microscale thermophoresis.

To confirm the interaction between rAPOE isoforms and LPS from *E. coli*, we conducted measurements of intrinsic fluorescence. This method detects shifts in the signal of aromatic amino acids, primarily tryptophan, in the protein, reflecting structural changes as a result of ligand interactions. The obtained data indicate that rAPOE4 exhibits a higher affinity for LPS (with a *K*_*D*_ of 0.6 ± 0.2) in comparison to rAPOE3 (with a *K*_*D*_ of 1.3 ± 0.1), as illustrated in Figure 3*A*. Similarly, microscale thermophoresis (MST), a highly sensitive technique for determination of interactions between proteins and ligands in solution, revealed interactions of fluorescence-labeled LPS-FITC with APOE3 (*K*_*D*_ of 1.10 ± 0.36 μM) and APOE4 (*K*_*D*_ of 0.35 ± 0.20 μM) (Fig. 3*B*).

### Formation of macromolecular complexes of APOE isoforms with endotoxin

**Figure 4:**
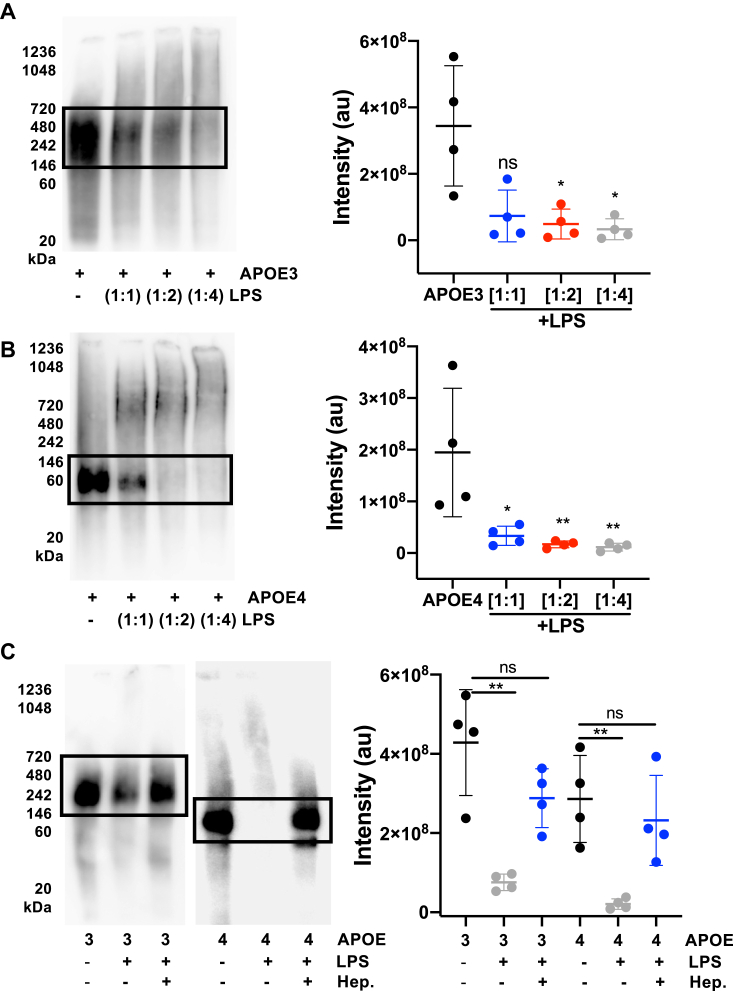
**Complex formation between APOE isoforms and LPS.** rAPOE3 (*A*), E4 (*B*), or both isoforms (*C*) (5 μM) were mixed with either Tris buffer, 10 μM LPS from *Escherichia coli*, or heparin. Samples were incubated for 30 min at room temperature and then analyzed by Western blot following Blue-Native gel. One representative image from four independent experiments is shown (n = 4). *C*, image analyses of Blue-Native gel images of rAPOE3 and rAPOE4 complexes with LPS from *E. coli* using ImageJ. Statistical analysis was performed using a one-way ANOVA with Dunnett’s multiple comparison tests, ∗*p* ≤ 0.05, ∗∗*p* ≤ 0.01, and ns = not significant. APOE, apolipoprotein E; LPS, lipopolysaccharide; rAPOE, recombinant APOE.

Our investigation aimed to determine whether rAPOE isoforms interact with LPS. To assess the difference between APOE isoforms, the proteins were subjected to LPS derived from *E. coli* and subsequently analyzed using Blue-Native (BN) PAGE and Western blot analysis, specifically targeting rAPOE (Fig. 4, *A* and *B*). We noted a significant shift toward higher molecular complexes when rAPOE4 was combined with LPS at a molecular ratio of 1:1. Furthermore, we observed a substantial reduction in the signal for rAPOE3 following LPS exposure at a molecular ratio of 1:2. This decrease in signal may be attributed to the formation of very high molecular weight complexes involving APOE, which are unable to enter the BN gel. Furthermore, we demonstrated the reversibility of complex formation between rAPOE isoforms and LPS by introducing heparin into the mixture at a 1:2 ratio between LPS and heparin (w/w) (Fig. 4*C*).

In addition, we discovered that both rAPOE isoforms can interact with LPS obtained from *P. aeruginosa*, resulting in the formation of high molecular weight complexes. However, this interaction did not occur in the presence of lipid A from *E. coli*, as indicated by no noticeable change in molecular weight of rAPOE4 (Fig. S2, *A* and *B*).

### All-atom MD simulations of APOE isoforms interacting with *E. coli* LPS

**Figure 5:**
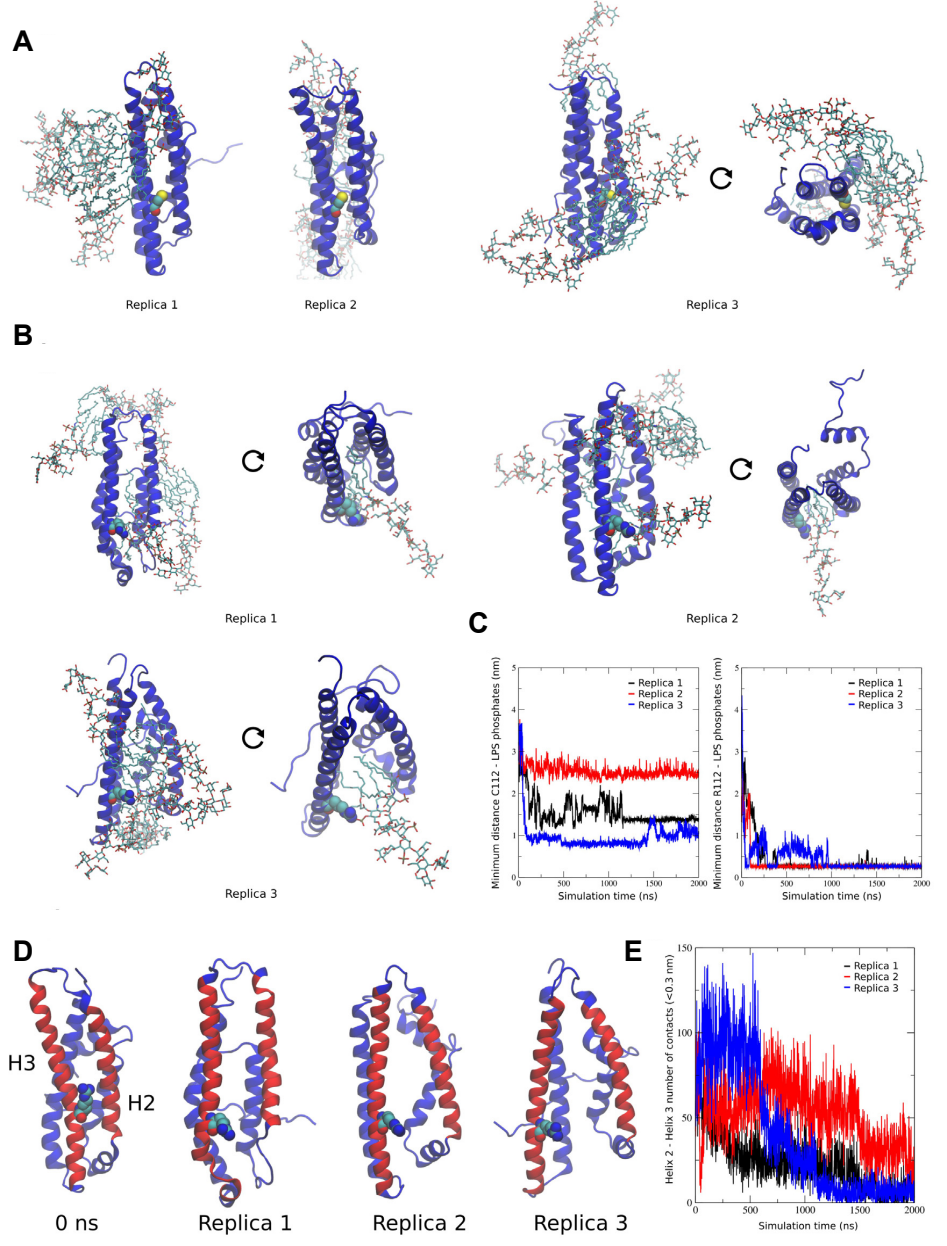
**MD simulations of APOE isoforms interacting with *Escherichia coli* lipopolysaccharide (LPS).** Final simulation snapshots of randomly placed LPS molecules around (*A*) APOE3 and (*B*) APOE4, run in triplicates of 2 μs each. *C*, minimum distance between residue C112 for APOE3 (*left*) or R112 for APOE4 (*right*) and LPS lipid A phosphate groups. *D*, initial (0 ns) and final simulation snapshots of all replicas of APOE4 showing open pocket located between helix 2 (H2) and helix 3 (H3). *E*, number of contacts between H2 and H3 over the simulation time for APOE4 system for all replicas. In all panels, protein is shown in *cartoon representation* in *blue*, whereas in (*D*), H2 and H3 are shown in *red*. Residue 112 is shown as *spheres*, whereas LPS is shown as *sticks* (carbon—*cyan*, oxygen—*red*, nitrogen—*blue*, phosphorus—*brown*, and sulfur—*yellow*). APOE, apolipoprotein E; MD, molecular dynamics.

Explicitly solvated atomic resolution MD simulations of APOE3 and APOE4 were conducted in the presence of *E. coli* LPS to assess potential interaction sites. Previous simulations of full-length APOE3 revealed that the C-terminal domain, which is responsible for host lipid–lipoprotein interactions, is highly dynamic and spontaneously dissociates from the N-terminal domain to expose the amphipathic antiparallel four-helix bundle N-terminal domain for ligand binding (7). Accordingly, we focused on the truncated variants of APOE3 and APOE4 (residues 1–167) to improve sampling. Five LPS molecules were randomly placed around either truncated APOE3 or truncated APOE4. Simulations of each system were run for 2 μs in triplicate, in each case starting from a different arrangement of LPS molecules around the APOE isoform. Across all simulations for both APOE constructs, LPS molecules were observed to spontaneously aggregate with one another as well as with the protein through hydrophobic interactions of the lipid tails that led to them wrapping around the protein surface (Fig. 5, *A* and *B*). Electrostatic interactions were also observed between the lipid A phosphate groups and basic residues of the protein, but in this context, significant differences were observed between APOE3 and APOE4 as a result of the substitution of the neutral cysteine for positively charged arginine at position 112 lying on helix 3 (H3). Thus, in the case of APOE3, in two simulation replicas, C112 was not involved in any interactions with LPS. In the third replica, some adsorption of LPS molecules around helix 2 (H2) and H3 was observed (Fig. 5*A* and Movie S1). In contrast, R112 of APOE4 was observed to establish long-lived electrostatic interactions with LPS phosphates consistently in all simulation replicas (Fig. 5*B* and Movie S2). This is supported by measurement of the minimum distance between the side chain of residue 112 and lipid A phosphate groups over the simulation time for each system (Fig. 5*C*). Strikingly, for APOE4, as a result of the anchoring of lipid A phosphates to R112, the acyl tails of LPS were subsequently observed to enter between H2 and H3 (Fig. 5*D*), resulting in dissociation of the two helices, as supported by the reduced number of contacts between them over time (Fig. 5*E*). This was in stark contrast with APOE3 where H2 and H3 remained bound to one another throughout all the 2 μs simulation replicas (Fig. S3). This is consistent with the reduced heat stability of APOE4 compared with APOE3 in the presence of LPS as well as its increased tendency to undergo structural changes. Furthermore, this suggests that APOE4 has a greater capacity for LPS adsorption and provides a molecular rationale for its higher LPS binding affinity compared with that of APOE3.

### Endotoxin induces aggregation of APOE isoforms

**Figure 6:**
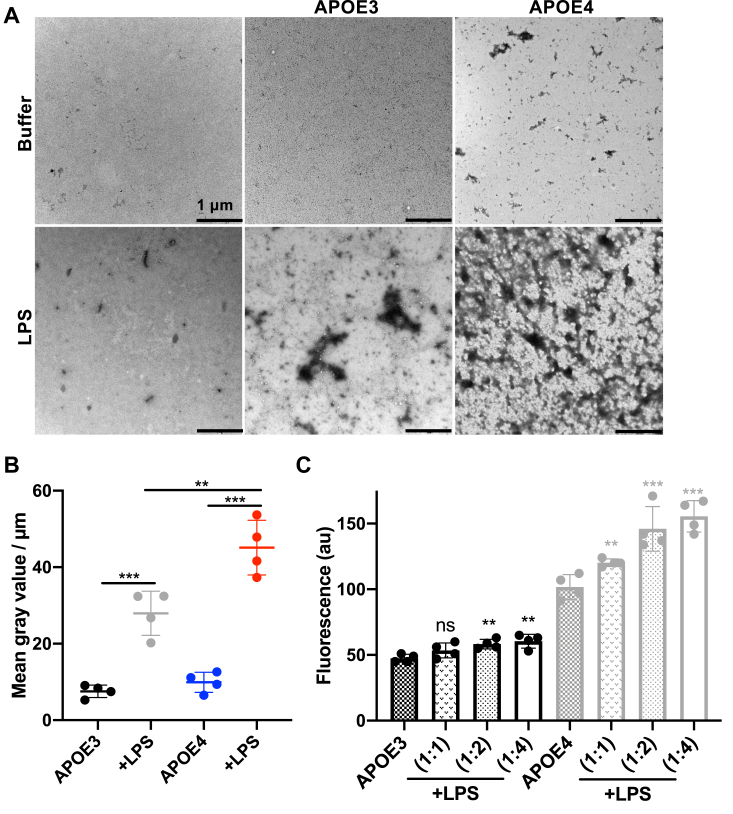
**APOE aggregation triggered by LPS.***A*, TEM-negative stain analysis revealed the presence of amorphous aggregates in both APOE isoforms exposed to LPS (10 μM). The images represent a representative example from three independent experiments. The scale bar corresponds to 1 μm. *B*, image analyses of TEM images of APOE3 and APOE4 complexes with LPS from *Escherichia coli* using ImageJ. *C*, in the ThT aggregation assay, a significant increase in ThT fluorescence was observed in both isoforms of APOE (5 μM) after the addition of 5, 10, and 20 μM of LPS from *E. coli*. No increase in ThT signal was seen in the presence of APOE3 and 5 μM of LPS compared with APOE4. Statistical analysis was performed using one-way ANOVA with Dunnett’s multiple comparison tests from four independent experiments (n = 4). ∗∗*p* ≤ 0.01 and ∗∗∗*p* ≤ 0.001. APOE, apolipoprotein A; LPS, lipopolysacharide; TEM, transmission electron microscopy; ThT, thioflavin T.

The findings from the shift assay were further corroborated through transmission electron microscopy (TEM) with negative staining, which demonstrated the formation of aggregates when rAPOE isoforms were exposed to LPS. Notably, rAPOE4 complexes were larger than those formed with APOE3 when mixed with LPS at a 1:1 M ratio (Fig. 6*A*). This difference in aggregate size was confirmed by image analysis, revealing a larger distribution of the mean complex area in samples containing rAPOE4 compared with rAPOE3 (Fig. 6*B*). To confirm the presence of protein aggregation, we utilized thioflavin T1 (ThT1) staining, a method commonly employed for identifying the β-sheet structures typically associated with amyloidogenic proteins. Our results indicated a rise in β-sheet content that was proportional to the concentration of LPS treatment in both rAPOE isoforms. Notably, rAPOE4 exhibited a higher β-sheet content, both in isolation and when exposed to LPS, compared with the rAPOE3 isoform (Fig. 6*C*).

To underscore the differential interaction of rAPOE isoforms, we also detected the formation of protein aggregates when rAPOE3 was challenged by LPS from *P. aeruginosa* or lipid A from *E. coli*, using TEM analysis. We observed an increase in protein aggregates when rAPOE3 was exposed to both LPS from *P. aeruginosa* and lipid A from *E. coli*. However, we did not observe the same effect in the protein aggregates of rAPOE4 when challenged by lipid A from *E. coli* compared with LPS from *P. aeruginosa* (Fig. S4).

**Figure 7:**
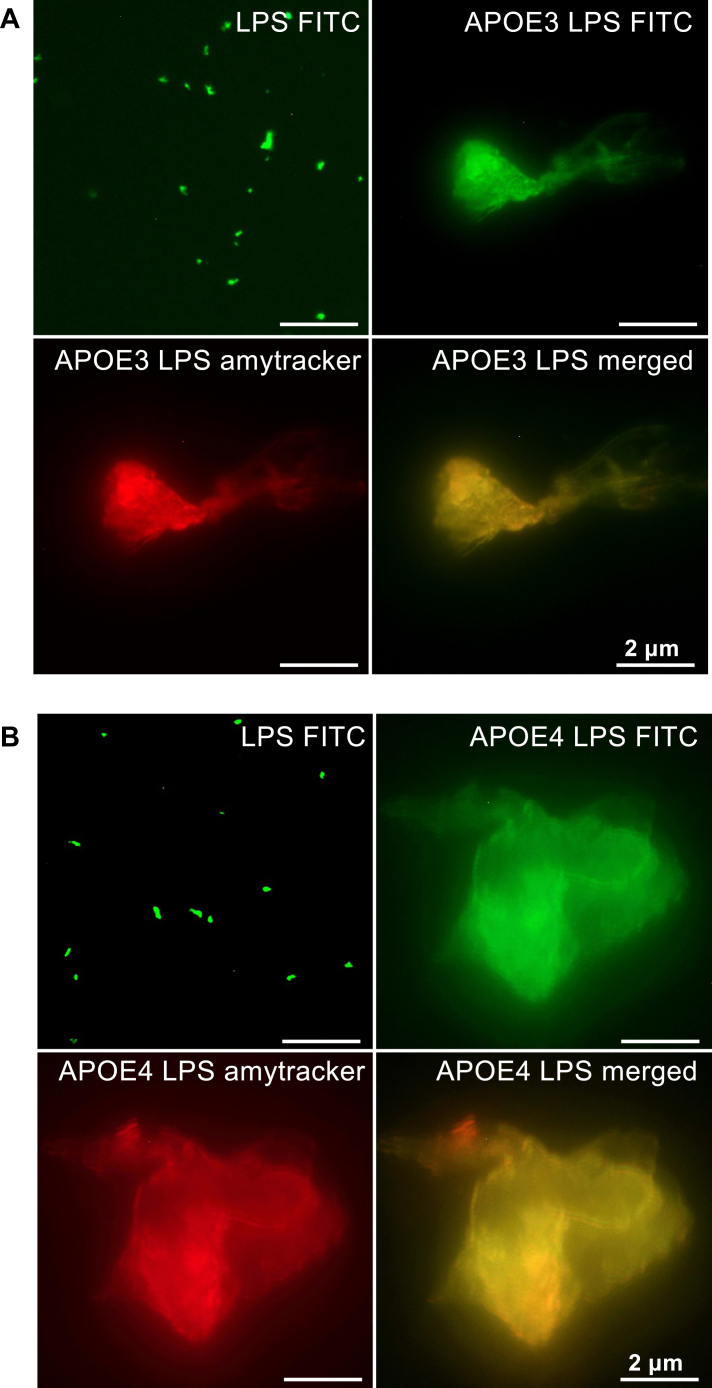
**Fluorescence microscopy revealed the presence of protein aggregates.** 5 μM of APOE3 (*A*) and APOE4 (*B*) stained with Amytracker 680 stain (*red*) and exposed to LPS-FITC (10 μM) (*green*). The provided images represent an example from three independent experiments. The scale bar corresponds to 2 μm. APOE4, apolipoprotein 4.

In addition to TEM, we utilized fluorescent microscopy imaging to visualize the formation and composition of protein aggregates in rAPOE isoforms challenged with LPS. These aggregates were stained with Amytracker and treated with LPS-FITC, allowing us to visualize the presence and distribution of LPS within the aggregates (Fig. 7, *A* and *B*). It is important to note that rAPOE isoforms alone did not exhibit the formation of aggregates (Fig. S5).

Using fluorescent microscopy, similar differences in aggregate size between the APOE3 and APOE4 were observed. Amytracker 680, a fluorescent tracer, provides high-quality visualization of protein aggregation and amyloids. With this fluorescent probe, an increase in fluorescence signal was detected in rAPOE4 compared with rAPOE3 (5 μM) exposed to 5 μM of *E. coli* LPS (Fig. 7). Buffer and LPS alone produced a very low fluorescence signal (Fig. S5). To study LPS presence within the aggregates, FITC-labeled LPS was utilized. Fluorescence microscopy imaging showed the presence of FITC label within the aggregates, which were stained by Amytracker 680. This staining combination confirmed the incorporation of FITC-LPS into the amyloid aggregates.

### Immunomodulatory effects of APOE3 and APOE4 isoforms during LPS challenge *in vitro* and *in vivo*

**Figure 8:**
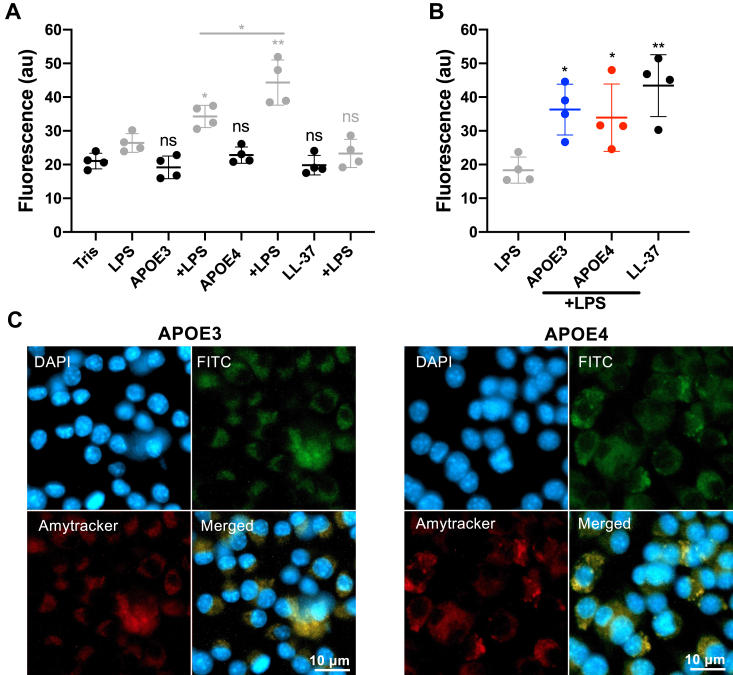
**Uptake effect of APOE isoforms *in vitro*.** A phagocytosis assay, conducted using the macrophage cell line RAW 264.7, revealed a significant increase in phagocytosis of protein aggregates in acute wound fluids stained with Amytracker 680 (*A*) and LPS-FITC (*B*). Statistical analysis was performed using one-way ANOVA with Dunnett’s multiple comparison tests from four independent experiments (n = 4). ∗*p* < 0.05, ∗∗*p* < 0.01, and ns = not significant. *C*, fluorescence microscopy imaging of protein aggregates was conducted using APOE isoforms pretreated with LPS (FITC, g*reen*) and stained with Amytracker 680 (*red*). RAW 264.7 cells were utilized for the experiment, and DAPI staining was applied to visualize the cell nuclei (*blue*). The images represent a representative example from three independent experiments. The scale bar corresponds to 10 μm. APOE, apolipoprotein E; DAPI, 4′,6-diamidino-2-phenylindole; LPS, lipopolysaccharide.

To investigate functional aspects among APOE isoforms concerning LPS-induced aggregation, we conducted a phagocytosis assay using fluorescence detection to delve deeper into the interplay between protein aggregates induced by LPS and the uptake by macrophages. Amytracker staining of the aggregates facilitated the monitoring of their uptake by macrophages. Our findings revealed a notable increase in the uptake of protein aggregates in rAPOE proteins challenged with LPS compared with LPS alone (Fig. 8*A*). Furthermore, the data indicate that uptake by the cells was significantly more pronounced for the LPS-treated E4 isoform when compared with the LPS-treated E3 isoform (Fig. 8*A*). In addition, we validated the internalization of protein aggregates by subjecting both rAPOE isoforms to FITC-labeled LPS (Fig. 8*B*). Results from both assays consistently demonstrated a significant augmentation in the uptake of protein aggregates in LPS-challenged rAPOE proteins in comparison to LPS alone. LL-37 was employed as a positive control in cell assays to investigate the immunomodulatory effects during LPS treatment, as shown in previous studies (12).

Moreover, we employed fluorescent microscopy to image macrophages exposed to Amytracker-stained amyloid-forming aggregates in rAPOE proteins with FITC-LPS. This technique allowed us to visualize the internalization of protein aggregates by macrophages, which were dual-labeled with Amytracker and FITC-labeled LPS (Fig. 8*C*).

**Figure 9:**
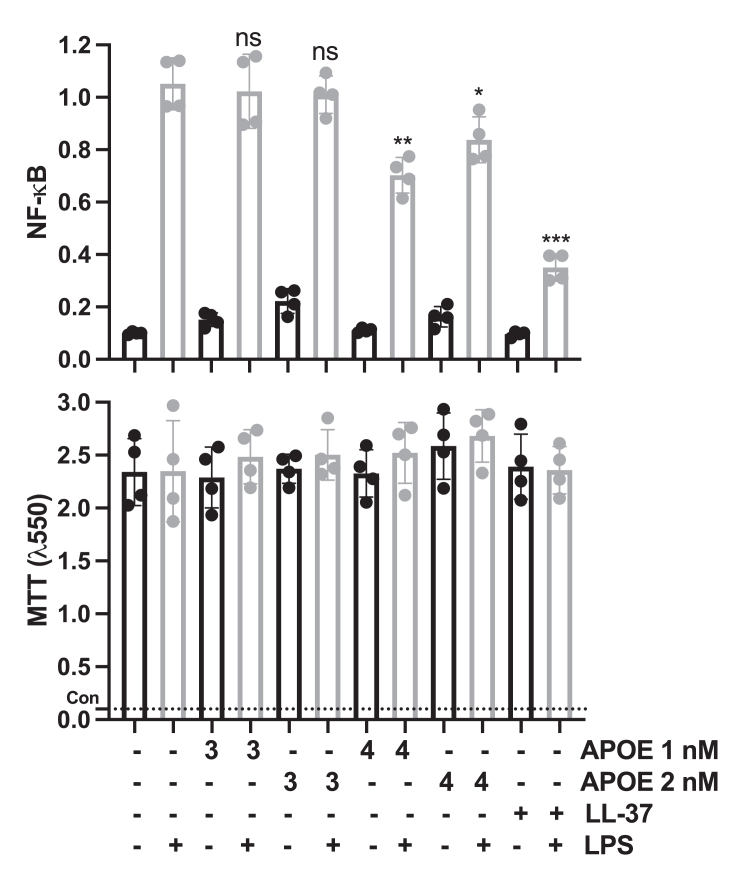
**Immunomodulatory effect of APOE isoforms *in vitro*.** THP-1-XBlue-CD14 cells underwent treatment with rAPOE3/4 (1 nM [1:1] and 2 nM [2:1]), LPS (10 ng/ml) from *Escherichia coli*, or a combination of both. rAPOE4 demonstrated a significant reduction in the activation of NF-κB/AP-1. MTT viability assay was employed to analyze the potential toxic effects of APOE on THP-1 cells. The *dotted line* (con) represents the positive control for dead cells. The mean values of four measurements ± their SD are displayed (n = 4). *P* values were determined using one-way ANOVA with Dunnett’s multiple comparison test. ∗*p* < 0.05, ∗∗*p* < 0.01, and ns = not significant. APOE, apolipoprotein E; LPS, lipopolysaccharide; MTT, 3-(4,5-dimethylthiazol-2-yl)-2,5-diphenyltetrazolium bromide; rAPOE, recombinant APOE.

Subsequently, we assessed NF-κB/AP-1 activation in THP-1-XBlue-CD14 reporter monocytes. The results indicated that the E4 isoform reduced LPS-induced NF-κB activation relative to E3 under identical experimental conditions (13). Notably, neither of the APOE isoforms exerted any influence on cell viability (Fig. 9).

**Figure 10:**
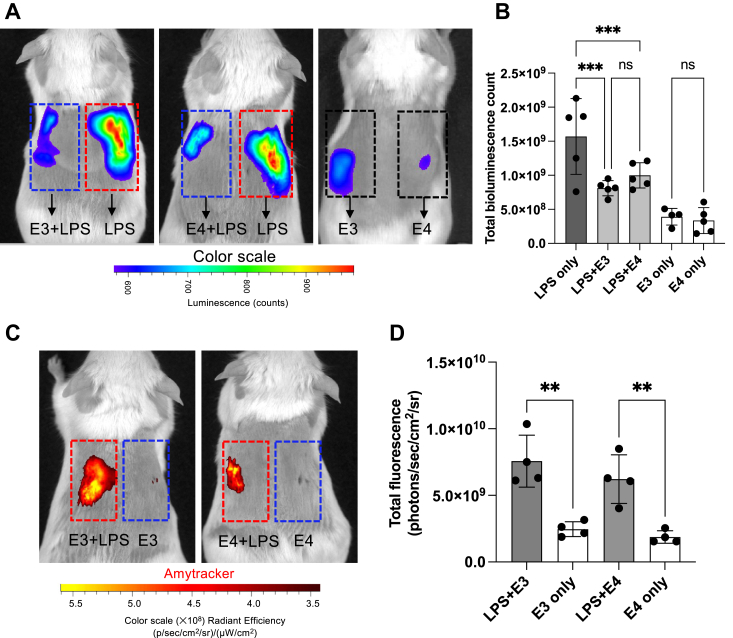
**Immunomodulatory effect of APOE isoforms *in vivo***. *A*, *In vivo* inflammation imaging by IVIS in NF-κB reporter mice. LPS (5 μM) was subcutaneously administered into either the *left* side or the *right* side of the mouse dorsum, with or without 100 μl of APOE. *In vivo* imaging was performed using an IVIS Spectrum bioimaging system 3 h after subcutaneous deposition. *B*, the bar chart shows the bioluminescence intensity emitted from these mice. *Dotted rectangles* show the region of interest. Data are presented as the means ± SD (n = 4–5). *C*, *in vivo* detection of APOE aggregates using IVIS imaging in fluorescence mode. APOE isoforms alone or pretreated with LPS were stained with Amytracker 680 (*red*). Preparation was subcutaneously administered into either the *left* side or the *right* side of the mouse dorsum, and fluorescence imagining was performed using IVIS. Representative heat map overlays obtained from emitted light are shown. *D*, bar chart shows the measured radiance emitted from the region of interest (*dotted rectangle*). Data are presented as the means ± SD (n = 4). ∗∗*p* ≤ 0.01, ∗∗∗*p* ≤ 0.001, and ns = not significant. APOE, apolipoprotein E, IVIS, *in vivo* bioimaging system; LPS, lipopolysaccharide.

We then explored whether APOE3/4 could mitigate local inflammation induced by LPS *in vivo*. To conduct this study, we employed the NF-κB-RE-Luc reporter mouse model and examined the impact of APOE isoforms on subcutaneous inflammation triggered by LPS (*E. coli*). LPS (5 μM) was subcutaneously administered into either the left side or the right side of the mouse dorsum, with or without 100 μl of APOE (5 μM). Following the injection of luciferin substrate, the luminescent signal indicative of NF-κB activation was captured using an *in vivo* bioimaging system (IVIS Spectrum) (Fig. 10*A*). Analysis of bioluminescent emissions revealed a notable reduction in NF-κB activation at the site administered with both APOE isoforms and LPS after 3 h, compared with LPS treatment alone (Fig. 10*B*). APOE alone did not result in a significant increase in NF-κB activation. Further, to confirm the presence of APOE aggregation, APOE isoforms alone or pretreated with LPS were stained with Amytracker 680 (red) and administered into either the left side or the right side of the mouse dorsum. Three hours after administration, fluorescence imaging was performed using IVIS (Fig. 10*C*). Results show detection of significantly higher amount of aggregates at the site administered with both APOE isoforms and LPS, compared with APOE isoforms alone (Fig. 10*D*).

## Discussion

In this study, we built upon our prior investigations into the antimicrobial and immunomodulatory activities of human APOE, which was purified from human plasma. Our earlier work demonstrated APOE's bactericidal capabilities against Gram-negative bacteria and its capacity to neutralize LPS (6, 7). In addition, we have documented that proteins within the apolipoprotein family, including APOE, which are found in acute wound fluids, exhibit the ability to aggregate when exposed to LPS. Moreover, such LPS-wound fluid protein aggregates can effectively mitigate the toxic effects associated with LPS (14).

In this current study, we reaffirmed that rAPOE3/rAPOE4s can bind to and aggregate LPS in an APOE isoform-specific manner. We also confirmed the antimicrobial efficacy against various strains of Gram-negative bacteria, specifically *P. aeruginosa* and *E. coli*. Correspondingly, peptides derived from the receptor and lipid-binding regions of APOE (specifically, residues 138–150 and 244–272) have been shown to possess antibacterial and immunomodulatory properties (15, 16).

LPS, a potent proinflammatory molecule found in Gram-negative bacteria, can cause excessive inflammation if unregulated. We unveil a simple yet highly efficient mechanism by which APOE isoforms aggregate and potentially alleviate the impact of LPS. Using mice and IVIS bioimaging, our study demonstrates that APOE-mediated LPS aggregation significantly reduces local inflammation.

This mechanism is particularly relevant in the context of neurodegenerative diseases, such as Parkinson's disease, Huntington's disease, and Alzheimer's disease (AD), where LPS and related aggregates have been identified (14, 17). For instance, in AD patients, APOE has been identified alongside amyloid-ß peptides (18). The analysis of brain amyloid imaging and cerebrospinal fluid biomarkers reveals the early presence of amyloid deposition in individuals carrying the APOE-ε4 genetic risk factor (19). It is proposed that LPS, often associated with infections or disruptions in the oral or gut microbiome, may enter the bloodstream and subsequently cross the blood–brain barrier, leading to an immune response and inflammation in the brain (20, 21).

If AD is indeed influenced by endotoxins, it stands to reason that genetic variations linked to AD might interact with endotoxins or processes related to endotoxin activity. The primary genetic risk factor for AD is the APOE isoform, with APOE2 offering a protective effect, APOE3 being viewed as neutral, and APOE4 having a detrimental impact on disease susceptibility (22). In addition to APOE, sequence variants of the LPS-receptor TLR4 (23) have also been linked to an increased risk of AD. These findings underscore additional genetic links between AD and the presence of endotoxins, further supporting the endotoxin hypothesis of neurodegeneration (21).

**Figure 11:**
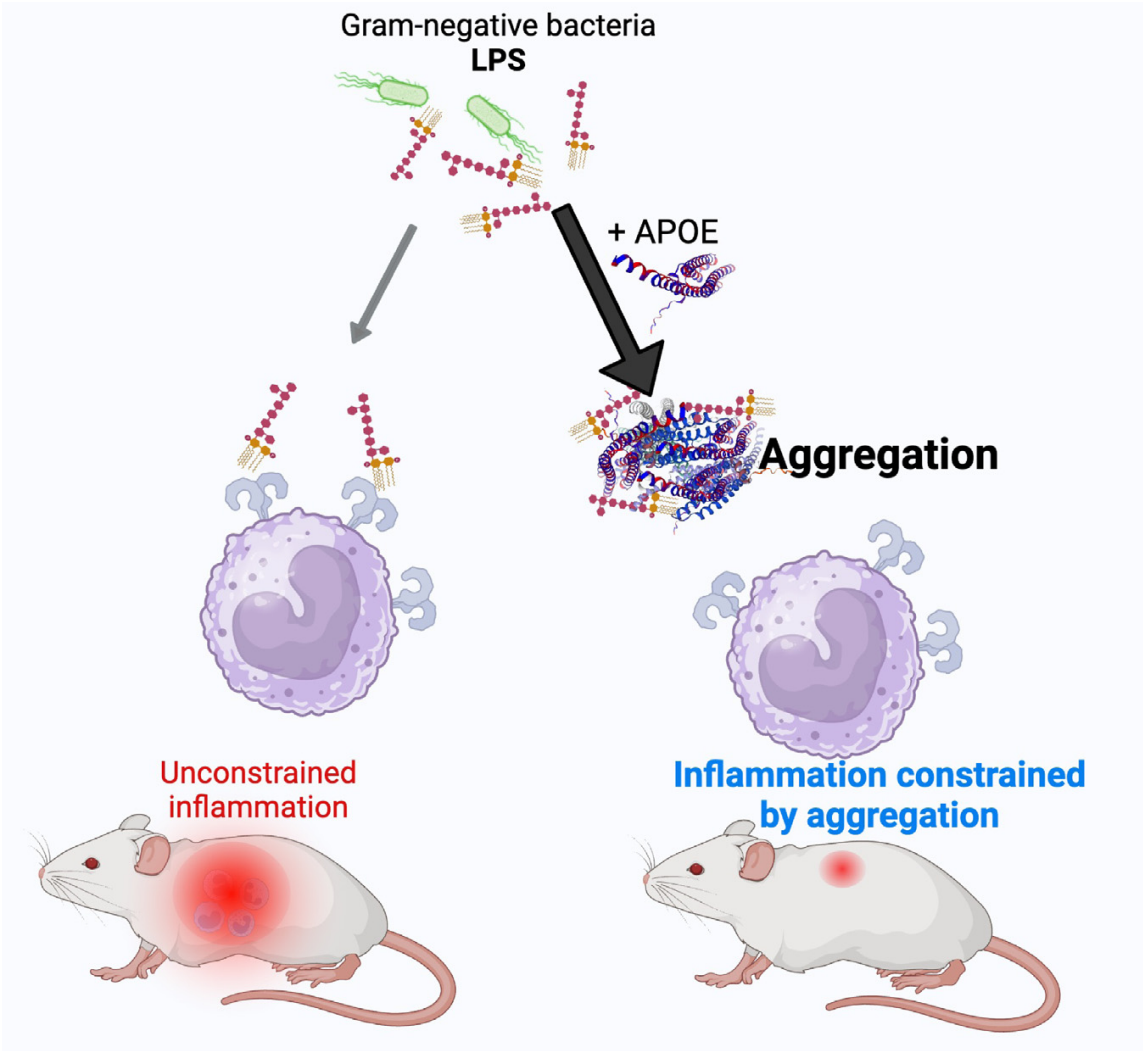
**Summary of APOE function**. APOE exhibits a neutralizing impact on Gram-negative bacterial infection. APOE, apolipoprotein E.

Research in rodents has shown that intravenous LPS administration results in a robust induction of serum APOE. APOE, in turn, directly interacts with LPS, facilitating its uptake and subsequent degradation by the liver. This phenomenon renders mice lacking APOE more susceptible to LPS toxicity, underscoring the crucial role of APOE in modulating the response to LPS (24). Figure 11 illustrates how proaggregation proteins like APOE promote the containment of LPS-induced inflammation.

From a pathophysiological standpoint, our discoveries concerning LPS-triggered APOE aggregation underscore the intricate connection between host defense mechanisms and amyloidogenesis, as corroborated by prior research (25, 26, 27, 28, 29). Thus, in instances of chronic inflammatory conditions characterized by an excessive activation of host defense, there may be a likelihood of abnormal aggregation.

Our *in vitro* data suggest that APOE4 may have a protective effect against infection-triggered LPS release. Remarkably, there exists epidemiological evidence pointing to an intriguing interplay between infectious burden and APOE ε4 carrier status, hinting at a potential safeguarding influence of APOE ε4 against infection and its enduring cognitive consequences. In a study involving an Amazonian cohort of forager-horticulturalists, individuals with high parasitic burdens who were APOE ε4 carriers demonstrated superior cognitive performance compared with their non-ε4 carrier counterparts (30). Another investigation within a sizable rural Ghanaian population suggested that APOE ε4 might confer protection against infection and support fertility, particularly among women exposed to elevated pathogen levels (31). These findings align with observations from various studies involving patients with chronic hepatitis, revealing that those possessing the APOE ε4 genotype exhibited a slower disease progression and more favorable outcomes (32, 33).

The exact mechanism by which APOE ε4 may offer protection against infection remains unclear. Nevertheless, there is evidence suggesting that antimicrobial properties may be associated with beta-amyloid deposits (34). According to the thrifty gene hypothesis (22), certain seemingly disadvantageous genotypes, including APOE ε4, might have conferred a survival advantage in high-income populations during preindustrial eras. Specifically, the potential protective effects of APOE ε4 against infection and support for fertility could have played a role in its selection in preindustrial populations characterized by a higher burden of infections and increased vulnerability to early mortality from childhood infections.

APOE isoforms, because of their natural aggregation properties, show potential as therapeutic agents against Gram-negative bacteria and endotoxins during infections. Previous studies, including recent findings, have demonstrated that APOE plays a role in LPS aggregation and clearance in wounds (6, 7, 14), further highlighting its potential in the development of anti-infective therapies. The study also provides valuable insights into how APOE-mediated protein aggregation contributes to LPS neutralization, thereby inhibiting activation of CD14/TLR4-mediated pathways. It can, however, not be excluded that APOE has additional context-specific effects on inflammatory processes *per se* not detected in the experimental settings used. Therefore, investigating these additional aspects, as well as how APOE isoforms compare to existing drugs, could drive the advancement of novel therapeutic approaches for combating bacterial infections.

Currently, several Food and Drug Administration–approved AMP drugs are available, including the cyclic lipopeptide daptomycin (Cubicin) (35), the linear peptide gramicidin (36), the cyclic lipopeptide polymyxin (37), and the cyclic peptide bacitracin (38) for both Gram-negative and negative bacterial infections. APOE's multifaceted roles in key biological processes make it an attractive target for drug development. Therapeutics leveraging its lipid transport, anti-inflammatory, and neuroprotective properties could address unmet needs in Alzheimer's disease, cardiovascular disorders, and infectious diseases.

In this context, it should be noted that *in vitro* studies offer valuable mechanistic insights under controlled conditions, whereas *in vivo* experiments provide a holistic and systemic perspective on biological processes. The differences between these approaches underscore the need to integrate findings from both types of experiments to achieve a more comprehensive understanding of APOE functions and enhance translational research outcomes. Furthermore, LPS experiments often reveal discrepancies between mouse models and human cells because of significant variations in LPS dose–response dynamics, species-specific immune responses, receptor signaling pathways, and cellular microenvironments (39).

While our *in vivo* results indicate a similar anti-inflammatory effect between APOE3 and APOE4, further studies are needed to fully understand the effects of these isoforms in host defense. Specifically, there may be subtle differences in how ApoE3 and ApoE4 interact with endotoxins and bacteria, influencing their ability to protect the host in the context of infection and inflammation. Future research should focus on exploring these potential differences by employing more detailed and specific experimental models that could uncover isoform-specific roles that are not evident from the current data. Moreover, the exact mechanism of APOE function, whether through direct bacterial killing or interference with bacterial metabolism, remains unclear. In-depth molecular studies are therefore necessary to clarify how APOE interacts with bacterial systems. Future research should also focus on human cell studies or investigations in more representative clinical settings.

## Conclusions

In conclusion, our study has provided evidence of the antimicrobial activity of lipid-poor/free rAPOE isoforms against various Gram-negative bacterial strains, including *E. coli* and *P. aeruginosa*. Notably, we observed that APOE4 exhibits a higher affinity for LPS from *E. coli* and forms bigger aggregates compared with APOE3, which may be explained at the molecular level by the LPS binding–induced conformational changes observed in our simulations. Further investigation into the endotoxin-scavenging abilities of APOE isoforms revealed notable differences in their effectiveness, with APOE4 showing more pronounced effects *in vitro* compared with APOE3. In addition, our *in vivo* data confirm that LPS-induced aggregation of APOE isoforms reduces local inflammation. Collectively, these findings suggest that the differential effects of APOE3 and E4 proteins may play an additional role in innate immunity during bacterial infections.

## Experimental procedures

### Bacterial strains

*E. coli* (25922) was purchased from American Type Culture Collection. *P. aeruginosa* clinical strain *P. aeruginosa* (PAO1) was kindly provided by Dr B. Iglewski (University of Rochester).

### Endotoxins

LPS from *E. coli* and *P. aeruginosa* were purchased from Sigma–Aldrich. Lipid A from *E. coli* was purchased from AH Diagnostics.

### Proteins and peptides

LL-37, the human cathelicidin AMP (sequence LLGDFFRKSKEKIGKEFKRIVQRIKDFLRNLVPRTES; 97% purity, acetate salt) was synthesized by Innovagen.

### Animals

BALB/c tg(NF-κB-RE-Luc)-Xen reporter male mice (10–12 weeks old), purchased from Taconic Biosciences, were used for all experiments. The animals were housed under standard conditions of light and temperature and had free access to standard laboratory chow and water.

### APOE protein purification

The genes-encoding h_apoE3 and E4 proteins were cloned into the commercial vector pET151/D-Topo with the N-terminal fusion tag (Invitrogen) according to the manufacturer’s instructions.

For protein expression, the plasmids with rAPOE4 and rAPOE3 genes were transferred to the BL21(DE3)pLysS *E. coli* cells (Invitrogen). One liter of cells in LB broth (Lennax, Sigma) was grown to midlog phase at 37 °C. These were then induced with 400 μM IPTG (Sigma) and incubated for an additional 3 h at 37 °C. The cells were harvested by centrifugation, and pellet stored for further protein purification. To purify the protein, we dissolved the pellet of bacteria in 8 M urea, 10 mM Tris buffer (pH 7.4) by sonication 10 × 10 s, and the sample was filtered through a 0.2 μm filter and then chromatographed. We employed affinity chromatography with a gravity column (Poly-Prep; Bio-Rad) filled with 1 ml of Ni–NTA Agarose (Invitrogen) and elution buffer 200 mM of imidazole. Next, the purified rAPOE was refolded by step-wise dialysis against 4, 2, 1, M urea and 10 mM Tris buffer (pH 7.4) using a dialysis cassette with a molecular size cutoff of 20 kDa (Slide-A-Lyzer; Thermo Scientific). To ensure that only full-length rAPOE was used for all experiments, we employed size-exclusion chromatography (ÄKTApurifier; GE Healthcare) with Superose 12 10/300 (GE Healthcare), mobile phase of 10 mM Tris (pH 7.4), and flow rate 0.5 ml/min. The sample was concentrated using centrifugal spin concentrators with a molecular size cutoff of 30 kDa (Millipore), and the protein concentration was determined using the NanoDrop (ND 1000; NanoDrop). Protein purity was confirmed by SDS-PAGE with Gel Code Blue Safe Protein (catalog no.: 1860983; Thermo Scientific), and Western blot analysis with mouse monoclonal antibody antihuman rAPOE (catalog no.: ab1906; abcam) was performed as described in the method for BN-polyacrylamide gel electrophoresis (see later) (1).

### Viable count assay

Potential antibacterial activity of APOE isoforms on *E. coli* and *P. aeruginosa* strains was explored by incubating one colony overnight in 5 ml of Todd–Hewitt medium. The next morning, t he bacterial culture was refreshed and grown to midlogarithmic phase (absorbance = 0.4 at 620 nm). The bacteria were then centrifuged, washed, and diluted 1:1000 in 10 mM Tris buffer at pH 7.4 to obtain an approximate concentration of bacteria amounting to 2 × 10^6^ colony-forming unit/ml. Next, 50 μl of bacterial suspension was incubated with 2 μM of rAPOE isoforms or buffer control (10 mM Tris buffer at pH 7.4) for 2 h at 37 °C. Two micromolars of LL-37 (used as a positive control), a human cathelicidin-derived AMP, which belong to the class of α-helical AMPs (40), was added. After 2 h, serial dilutions of the samples were plated on Todd–Hewitt agar plates, incubated overnight at 37 °C, and followed by colony counting the next day (6).

### Fluorescence microscopy

#### Live/dead bacteria

*E. coli* and *P. aeruginosa* viability in the aggregates was assessed by using LIVE/DEAD BacLight Bacterial Viability Kit (Invitrogen, Molecular Probes). Bacterial suspensions were prepared as described previously for VCA. Bacterial strains were treated with 2 μM rAPOE3 and rAPOE4, 2 μM LL-37, or 10 mM Tris at pH 7.4. After a 1-h incubation time at 37 °C, samples were mixed 1:1 with the dye mixture, followed by incubation for 15 min in the dark at room temperature. The dye mixture was prepared according to the manufacturer's protocol, that is, 1.5 μl of component A (SYTO-9 green-fluorescent nucleic acid stain) and 1.5 μl of component B (red-fluorescent nucleic acid stain propidium iodide) were dissolved in 1 ml of 10 mM Tris at pH 7.4. Five microliters of stained bacterial suspension was trapped between a slide and an 18 mm square coverslip (7).

We used Amytracker 680 (Ebba Biotech) staining to visualize amyloid formation of proteins. We incubated rAPOE (5 μM) with LPS-FITC (5 μM), from *E. coli* (Sigma–Aldrich) for 30 min at 37 °C. The samples were subsequently incubated with 50 μl of Amytracker 680 (1000× dilution from the purchased stock solution) in the tube for an additional 30 min of incubation at room temperature. Next, the samples were washed, transferred onto glass slides coated with l-lysine (SIGMA), and mounted on microscope slides with fluorescent mounting media (Dako North America) (14).

#### Thioflavin T assays

The formation of amyloids was assessed using ThT dye, known for its selective binding to the β-sheet structures characteristic of amyloidogenic proteins and peptides. To explore the relationship between concentration and aggregation, we incubated rAPOE3/4 at a concentration of 5 μM and LPS from *E. coli* at 5, 10, and 20 μM in a buffer solution containing 10 mM Tris at pH 7.4 for 30 min at 37 °C prior to measurements. Subsequently, 200 μL of these samples were incubated with 100 μM ThT for 15 min in a light-protected environment (ThT stock solution was 1 mM and stored in the dark at 4 °C). We measured ThT fluorescence using a VICTOR3 Multilabel Plate Counter spectrofluorometer (PerkinElmer) at an excitation of 450 nm, with excitation and emission slit widths of 10 nm. The baseline (10 mM Tris [pH 7.4] and LPS) was subtracted from the signal of each sample (7, 14).

#### Transmission electron microscopy

The APOE isoforms were observed using TEM with a Jeol Jem 1230 microscope from Jeol. Prior to and following incubation with LPS (*E. coli* and *P. aeruginosa*), lipid A or a buffer, negative staining was applied. Images of endotoxins at a concentration of 5 μM, both with and without the presence of rAPOE isoforms at 5 μM, were captured after a 30-min incubation at 37 °C. For the mounted samples, 10 different fields of view were examined on a grid with a pitch of 62 μm, and this analysis was conducted across three independent sample preparations. Samples were adsorbed onto carbon-coated grids (copper mesh, 400) for 60 s and stained with 7 μl of 2% uranyl acetate for 30 s. The grids were rendered hydrophilic *via* glow discharge at low air pressure. The size of aggregates was analyzed as the mean of gray value/μm ± SD by ImageJ 1.52k, after all the images were converted to 8 bit and the threshold was manually adjusted (6, 14).

#### BN-polyacrylamide gel electrophoresis and Western blot

Twenty-one microliters of rAPOE3/4 solution at a concentration of 5 μM was combined with either a 10 mM Tris buffer alone or LPS at a concentration of 5, 10, and 20 μM (1:1, 1:2. and 1:4 M ratios) or heparin (200 μg/ml). The mixtures were incubated for 30 min at 37 °C before being combined with a loading buffer (4× Loading Buffer Native Gel, catalog no.: BN2003; Life Technologies), and subsequently, 28 μl was loaded onto 4 to 16% Bis–Tris Native Gels (catalog no.: BN1002BOX; Life Technologies). Samples were run in parallel with a marker (Native Marker Unstained Protein Standard, catalog no.: LC0725; Life Technologies) at 150 V for 100 min. Gels were run in duplicates for each experiment: one for gel analysis after destaining from Coomassie and subsequent staining with Gel Code Blue Safe Protein (catalog no.: 1860983; Thermo Scientific), whereas the other was transferred to a 0.2 μm polyvinylidene fluoride membranes (Trans Blot Transfer Pack, catalog no.: 1704156; Bio-Rad) *via* a Trans Turbo Blot system (Bio-Rad). Thereafter, the membrane was destained with 70% ethanol and blocked with 5% milk in 1× PBS–Tween (PBS–T) for 30 min at room temperature. The membrane was incubated with mouse monoclonal antibody antihuman rAPOE (catalog no.: ab1906; abcam), at a concentration of 1 μg/ml diluted in 1% fat-free milk in 1× PBS-T, overnight at 4 °C. APOE and its high-molecular weight complexes were then detected using a secondary rabbit antimouse polyclonal antibody that was conjugated to horseradish peroxidase conjugate (catalog no.: P0260; Dako) (diluted 1:1000 in 1× PBS-T complemented with 5% milk) after incubation for 60 min at room temperature. PBS-T was used to wash the membrane after each step (3 × 10 min), and the last wash after the secondary antibody was performed four times. The bands were revealed by incubating the membrane in the developing substrate (Super Signal West Pico PLUS Chemiluminescent Substrate, catalog no.: 34580; Thermo Scientific). The signal was acquired by a Chemi-Doc (Bio-Rad) system (6, 7). The images were analyzed using ImageLab 6.1 (Bio-Rad; 2020) to detect the intensity of Western blot signals, with background signals subtracted from each individual measurement. All the experiments were performed at least three times.

#### Fluorescence spectroscopy

The emission fluorescence spectra of tryptophan in rAPOE3/4 were measured between 300 and 450 nm, following excitation at 280 nm. Intrinsic fluorescence of 5 μM rAPOE isoforms (10 mM Tris at pH 7.4) was measured in a 3 × 3 mm quartz cuvette using a Jasco J-810 spectropolarimeter equipped with an FMO-427S fluorescence module, a scan speed of 200 nm/min, and a 2-nm slit width. The interactions between rAPOE and bacterial ligands were performed by measuring intrinsic fluorescence of protein at 25 °C, after incubation with ligands (5 μM). Then *K*_*D*_ was calculated from the spectra shift of l_max_ as a function of the concentration of the bacterial ligands, after subtraction of the signal obtained for the protein alone. The results are expressed as an average of three independent experiments ± standard error of the mean (6).

#### Circular dichroism

CD measurements were performed using a Jasco J-810 spectropolarimeter equipped with a Jasco CDF-426S Peltier that was set to 25 °C. Proteins were diluted to 5 μM in 10 mM Tris at pH 7.4 and incubated with or without 5, 10, and 20 μM LPS (1:1, 1:2, and 1:4 M ratios) from *E. coli*. Measurements were performed after a 5-, 15-, or a 30-min incubation at room temperature in a 0.1 cm quartz cuvette. Scans were measured over the far-UV wavelength interval 200 to 260 nm, with a scan speed of 20 nm/min. An average of five scans for each sample was collected. The baseline (10 mM Tris buffer alone or with different ligands) was subtracted from the spectrum of each sample for normalization (6).

#### Microscale thermophoresis

We conducted MST analysis using the NanoTemper Monolith NT.115 apparatus from Nano Temper Technologies. Mixing a constant amount of 0.5 μM of LPS-FITC with increasing concentrations of rAPOE proteins in Tris buffer (10 mM, pH 7.4) preceded the analysis. Subsequently, 10 μl of each sample was loaded into standard glass capillaries (Monolith NT Capillaries, Nano Temper Technologies), and MST analysis was performed with the light-emitting diode and infrared laser both set at 80%. The *K*_*D*_ constants were determined using MST software MO Affinity Analysis, version 2.2.4 (41).

#### MD simulations

Initial coordinates of APOE3 were obtained from the Protein Data Bank (code: 2L7B (42)). The truncated version of APOE3, that is the N-terminal domain (residues 1–167), was modeled as described previously (7). The APOE4 protein was modeled by introducing the R112S mutation using the CHARMM-GUI web server (43). The CHARMM36m (44) forcefield and TIP3P explicit water model (45) were used, and ionization states were assigned assuming neutral pH. In all systems, the protein was placed in the center of a cubic box of dimensions 10 × 10 × 10 nm^3^. In each system, five *E. coli* LPS molecules were randomly placed around the protein surface. Simulations were run in triplicate for APOE3 and APOE4, with different random arrangements of LPS molecules in each case. This resulted in six systems in total. LPS molecules corresponded to the most frequently occurring species of *E. coli* and were composed of lipid A with inner and outer core sugars. Approximately 30,000 water molecules were added to each system. Magnesium chloride salt was added at a concentration of ∼150 mM, on top of neutralizing the overall system charge. Energy minimization was performed for each system using steepest descents for ≤5000 steps with a 0.01 nm step size. Successive equilibrations in the *NVT* and *NPT* ensembles were performed for 10 ns in total with position restraints applied to protein backbone atoms and lipid A phosphate groups. All unrestrained production simulations were run for 2 μs each in the NPT ensemble, using GROMACS2021.4 (46). Equations of motion were integrated *via* the Verlet leapfrog algorithm with a 2 fs time step. All bonds connected to hydrogens were constrained with the LINCS algorithm. The cutoff distance was 1.2 nm for the short-range neighbor list and van der Waals interactions, with forces smoothly switched to zero between 1.0 and 1.2 nm. The Particle Mesh Ewald method (47) was applied for long-range electrostatic interactions with a 1.2 nm real-space cutoff. The velocity rescaling thermostat was used to maintain the temperature at 310 K. Pressure was maintained at 1 bar using the Parinello–Rahman (48) barostat. All simulations were performed on the *National Supercomputing Centre*, Singapore (https://www.nscc.sg) using two nodes containing 128 CPUs (AMD EPYC 7713 64-Core processor) each. Snapshots were taken using Visual Molecular Dynamics (49).

#### Uptake assay

The RAW 264.7 macrophage cell line (passages 4, 5, 6, and 7), cultured in Dulbecco's modified Eagle's medium (DMEM) supplemented with 10% (v/v) fetal bovine serum and 1% (v/v) Antibiotic-Antimycotic solution, was seeded overnight in 96-well tissue culture plates (8 × 10^4^ cells per well) at 37 °C in a 5% CO_2_ atmosphere. APOE isoforms (50 μl, 100 nM) were mixed with and without LPS (100 nM, sourced from *E. coli*, Sigma–Aldrich). One set of experiments involved incubation with Amytracker 680 (1000× dilution from the stock solution) for 30 min at room temperature. The premixed samples were then added to the adherent RAW 264.7 cells for 1 h in DMEM. Another set of experiments utilized LPS-FITC (100 nM, from *E. coli*, Sigma–Aldrich), which was directly added together with rAPOE (100 nM) isoforms to the cells without preincubation (14).

For phagocytosis assessment, the cells were exposed to the specimens for 1 h at 37 °C, followed by two rinses with DMEM. Fluorescence levels were quantified using a Spectramax M2 Multilabel Plate Counter spectrofluorometer (Molecular Devices) with excitation/emission wavelengths set at 620/640 nm. The initial uptake from the media alone was subtracted from the signal of each sample.

#### NF-κB activity assay

The activation of NF-κB/AP-1 in THP-1-XBlue-CD14 reporter monocytes was assessed after 20 to 24 h of incubation following the manufacturer's protocol (InvivoGen). In brief, cells at a concentration of 1 × 10^6^ cells/ml in RPMI were plated in 96-well plates (180 μl) and exposed to peptides (LL-37 1 μM and rAPOE3/4 0.5, 1, and 2 nM), LPS (*E. coli*, 1 nM), or a combination overnight at 37 °C, 5% CO_2_, in a total volume of 200 μl. The next day, NF-κB/AP-1 activation was evaluated by measuring the secretion of embryonic alkaline phosphatase. Specifically, 20 μl of the cell supernatant was transferred to 96-well plates, and 180 μl of Quanti-Blue was added. After a 2 h incubation at 37 °C, absorbance was measured at 600 nm using a VICTOR3 Multilabel Plate Counter spectrofluorometer.

#### MTT viability assay

For the MTT (3-(4,5-dimethylthiazol-2-yl)-2,5-diphenyltetrazolium bromide) viability assay, sterile filtered MTT solution (Sigma–Aldrich) at a concentration of 5 mg/ml in PBS was stored in the dark at −20 °C until use. Subsequently, 20 μl of the MTT solution was added to the remaining overnight culture of THP-1-XBlue-CD14 reporter monocytes from the aforementioned NF-κB activity assay in 96-well plates, followed by incubation at 37 °C (as described previously). After a 2-h incubation at 37 °C, the supernatant was removed, and the blue formazan product generated in cells was dissolved by adding 100 μl of dimethyl sulfoxide (100%) to each well. The plates were gently shaken for 10 min at room temperature to dissolve precipitates, and absorbance was measured at 550 nm using a VICTOR3 Multilabel Plate Counter spectrofluorometer.

#### Mouse inflammation model

We employed BALB/c tg(NF-κB-RE-Luc)-Xen reporter male mice (14–15 weeks old; Taconic Biosciences) in a mouse inflammation model to investigate the impact of rAPOE isoforms on inflammation. One hundred microliters of LPS (*E. coli*, 10 μM) and rAPOE (5 μM) was preincubated for 30 min at room temperature with Amytracker 680 (50× dilution from the purchased stock solution). Hair from the dorsum of the mice was shaved and cleaned. Under isoflurane anesthesia (4% for induction and 2% for maintenance), 50 μl of the sample was subcutaneously injected at one side of the dorsum. At other side of the dorsum, 50 μl of LPS or APOE alone was injected (5 μM). The mice were transferred to individually ventilated cages. One hundred microliters of d-luciferin (PerkinElmer, 150 mg kg^−1^ body weight) substrate was intraperitoneally injected 15 min before IVIS imaging (14). Imaging was conducted 3 h after the subcutaneous administration. At the same time, to detect aggregates, IVIS imaging was performed under fluorescence mode. The bioluminescence or fluorescence emission was detected and quantified using Living Image 4.0 Software (PerkinElmer).

### Statistical analysis

The graphs of VCA, *K*_*D*_ constants, ThT assay, TEM analysis, BN gel analyses, bacterial/cell assays, and IVIS analyses are presented as the mean ± SD from at least three independent experiments. We assessed differences in these assays using one-way ANOVA with Dunnett’s multiple comparison tests or *t* test. All data were analyzed using GraphPad Prism (GraphPad Software, Inc). In addition, *p* values less than 0.05 were considered to be statistically significant (∗*p* < 0.05, ∗∗*p* < 0.01, ∗∗∗*p* < 0.001, and ∗∗∗∗*p* < 0.0001).

### Ethics statement

All animal experiments were performed according to the Swedish Animal Welfare Act SFS 1988:534 and were approved by the Animal Ethics Committee of Malmö/Lund, Sweden (permit numbers: M5934-19 and M8871-19). Animals were kept under standard conditions of light and temperature and water ad libitum.

## Data availability

The data will be shared upon request by contacting Jitka Petrlova *via* email at jitka.petrlova@mau.se.

## Supporting information

This article contains supporting information.

## Conflict of interest

The authors declare that they have no conflicts of interest with the contents of this article.
