# Supplementary material for: Apolipoprotein E3 and E4 isoforms exhibit differing effects in countering endotoxins
Source: J Biol Chem. 2025 Jan 27;301(3):108236. doi: 10.1016/j.jbc.2025.108236 (PMC11879696; doi:10.1016/j.jbc.2025.108236)
Supplement: Supporting information [file mmc3.docx]

Supporting Information

**Apolipoprotein E3 and E4 isoforms exhibit differing effects in countering endotoxins**

Manoj Puthia^1#^, Jan K. Marzinek^2#^, Katerina Vesela^3,4#^, Axel Larsson^1^, Artur Schmidtchen^1,5^, Peter J. Bond^2,6^ and Jitka Petrlova^7*^

^1^Division of Dermatology and Venereology, Department of Clinical Sciences, Lund University, SE-22184 Lund, Sweden, ^2^Bioinformatics Institute (BII), Agency for Science, Technology and Research (A*STAR), 30 Biopolis Street, #07-01 Matrix, Singapore 138671, Republic of Singapore, ^3^ BIOCEV, First Faculty of Medicine, Charles University, Průmyslová 595, 252 50 Vestec, Czech Republic, ^4^ Department of Paediatrics and Inherited Metabolic Disorders, First Faculty of Medicine, Charles University and General University Hospital in Prague, Ke Karlovu 455/2, 128 08 Prague, Czech Republic, ^5^Dermatology, Skåne University Hospital, SE-22185 Lund, Sweden, ^6^Department of Biological Sciences National University of Singapore, 14 Science Drive 4, Singapore 117543, ^7^Department of Biomedical Science, Faculty of Health and Society, Malmö University, SE-205 06 Malmö, Sweden

^#^ M.P., J.K.M. and K.V. contributed equally to this work

*To whom correspondence should be addressed: Jitka Petrlova, Division of Dermatology and Venereology, Department of Clinical Sciences, Lund University, Sölvegatan 19, Lund, SE-22184, Sweden; Tel.: +46 462223063; E-mail: [jitka.petrlova@mau.se](mailto:jitka.petrlova@mau.se)

**Running title**: Anti-endotoxin effects of apolipoprotein E isoforms

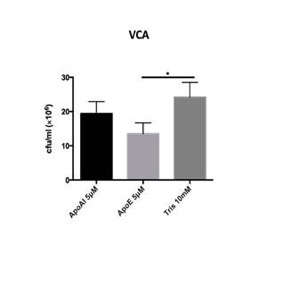

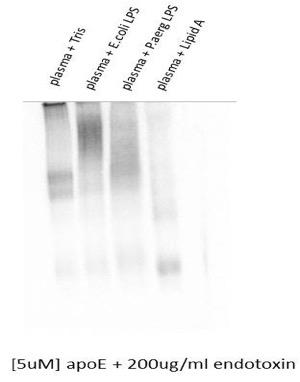

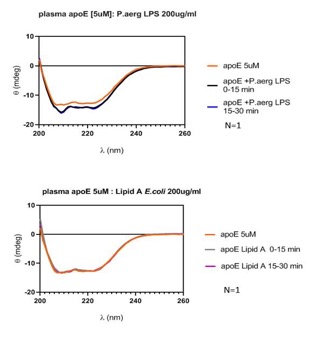

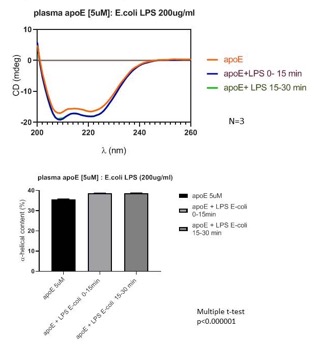
**Fig S1.**

**1B**

**2**

**3B**

**3A**

**Changes in the secondary structure of APOE isoforms upon temperature gradient.** CD spectra measured under differnet temperature conditions between 20 and 90 ºC for both rAPOE3 **(A)** and rAPOE4 **(B)**. Figures show representative spectra from at least four independent experiments (n =4).


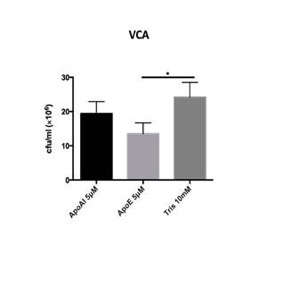

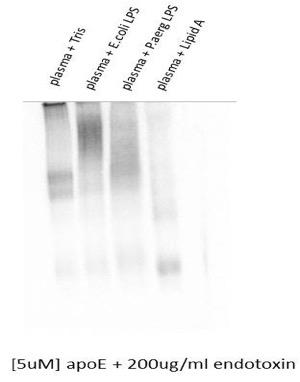

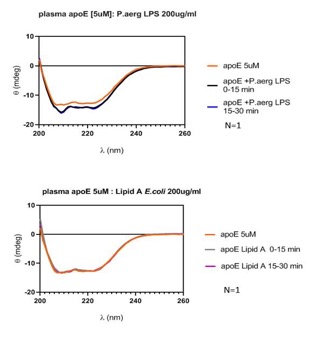

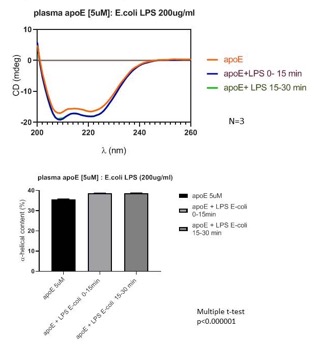
**Fig S2.**

**1B**

**2**

**3B**

**3A**

**Complex formation between APOE isoforms and LPS. A)** rAPOE3 and E4 (5 µM) was mixed with either Tris buffer or LPS from *E. coli* or *P. aeruginosa* (denoted as LPS_Ec_ and LPS_Pa,_ respectively), or with Lipid A from *E. coli* (LipA). Samples were incubated for 30 min at RT and then analyzed by western blot following Blue Native gel. One representative image from four independent experiments is shown (n=4). **B**) Image analyses of Blue Native gel images of APOE3 and APOE4 complexes with endotoxins using ImageJ. Statistical analysis was performed using a one-way ANOVA with Dunnett’s multiple comparison tests, ** = p ≤ 0.01, *** = p ≤ 0.001, and ns = not significant.

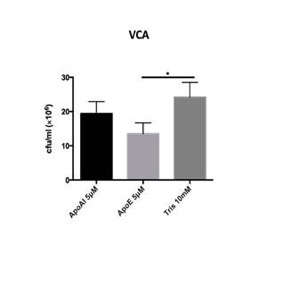

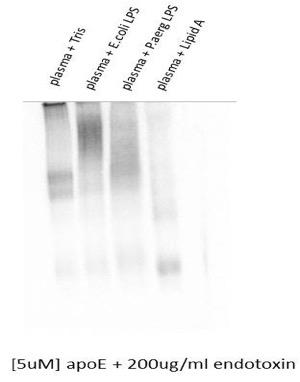

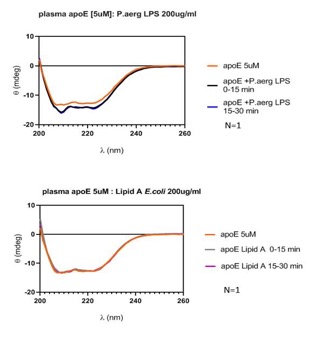

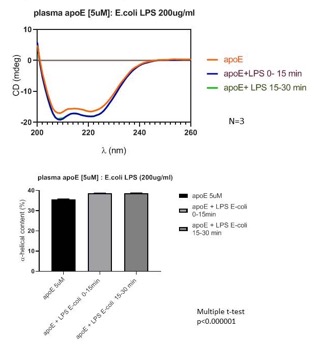
**Fig S3.**

**1B**

**2**

**3B**

**3A**

Number of contacts between helix 2 (H2) and helix 3 (H3) over the simulation time for APOE3 system for all replicas.

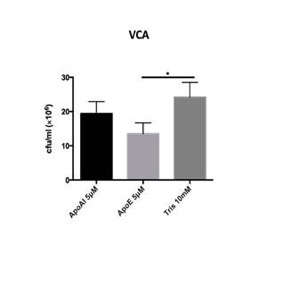

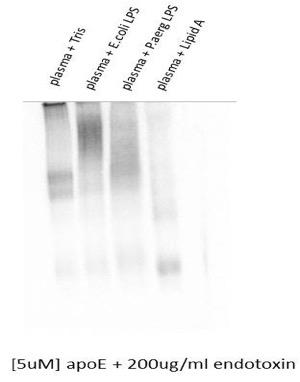

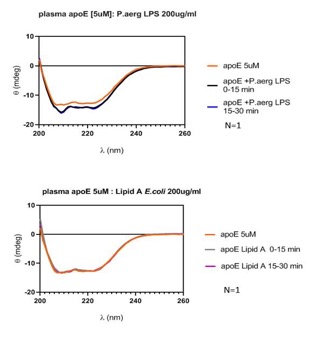

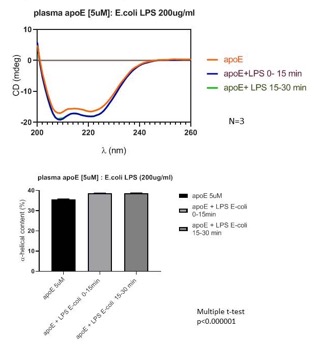
**Fig S4.**

**1B**

**2**

**3B**

**3A**

**Endotoxins induce APOE isoforms aggregation.** TEM analysis of rAPOE3 and E4 (5 µM), was mixed with either Tris buffer or LPS from *P. aeruginosa* (LPS_Pa_), or with Lipid A from *E. coli* (LipA).

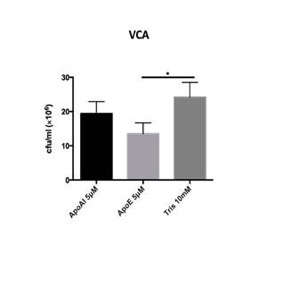

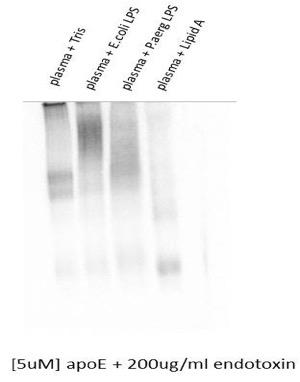

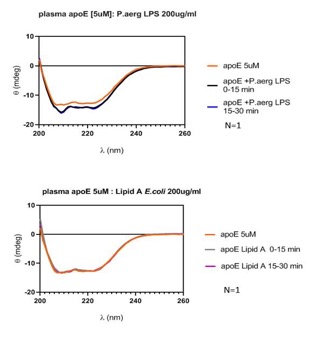

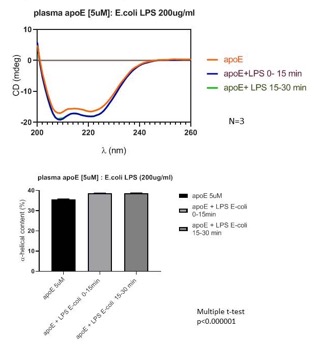
**Fig S5.**

**1B**

**2**

**3B**

**3A**

**Fluorescence microscopy unveiled the limited presence of protein aggregates.** 5 μM of rAPOE3 and rAPOE4 stained with Amytracker 680 stain (red). The provided images represent an example from three independent experiments. The scale bar corresponds to 2 μm.

**Movie S1.**

**MD simulations of LPS molecules spontaneously binding to truncated APOE3.** Protein is shown in blue cartoon representation, residue C112 side chain as spheres, while LPS molecules are shown in licorice representation (cyan – carbon; red – oxygen; blue – nitrogen; brown – phosphorus; sulphur – yellow).

**Movie S2.**

**MD simulations of LPS molecules randomly binding to truncated APOE4.** For clarity only one LPS molecules is shown. Protein is shown in blue cartoon representation with H2 and H3 in red. Residue R112 side chain is shown as spheres, while LPS molecules are shown in licorice representation (cyan – carbon; red – oxygen; blue – nitrogen; brown – phosphorus; sulphur – yellow).
